# *In vitro* activity of taurolidine against clinical *Candida auris* isolates: relevance to catheter-related bloodstream infections

**DOI:** 10.1128/aac.00381-24

**Published:** 2024-06-12

**Authors:** Bruce E. Reidenberg, Stephen G. Jenkins, Jared L. Crandon, Elizabeth Hurlburt, Xing Tan, Paul R. Rhomberg, S. J. Ryan Arends, Antony Pfaffle

**Affiliations:** 1 Department of Pharmacology, Weill Cornell Medicine, New York, New York, USA; 2 Division of Infectious Diseases, Department of Medicine, Weill Cornell Medicine, New York, New York, USA; 3 Department of Pathology and Laboratory Medicine, Weill Cornell Medicine, New York, New York, USA; 4 CorMedix Inc., Berkeley Heights, New Jersey, USA; 5 Element Iowa City (JMI Laboratories), North Liberty, Iowa, USA; University of Iowa, Iowa City, Iowa, USA

**Keywords:** taurolidine, *Candida auris*, catheter-related bloodstream infection, CLABSI

## Abstract

*Candida auris* is an evolving and concerning global threat. Of particular concern are bloodstream infections related to central venous catheters. We evaluated the activity of taurolidine, a broad-spectrum antimicrobial in catheter lock solutions, against 106 *C*. *auris* isolates. Taurolidine was highly active with a MIC_50_/MIC_90_ of 512/512 mg/L, over 20-fold lower than lock solution concentrations of ≥13,500 mg/L. Our data demonstrate a theoretical basis for taurolidine-based lock solutions for prevention of *C. auris* catheter-associated infections.

## INTRODUCTION

*Candida auris* is a highly concerning, emerging global health threat characterized by extensive drug resistance, invasive infections, and high morbidity and mortality (https://www.cdc.gov/candida-auris/tracking-c-auris/?CDC_AAref_Val=https://www.cdc.gov/fungal/candida-auris/tracking-c-auris.html) (1, 2). Resistance to available antifungals continue to increase, with the emergence of pan-resistant isolates and echinocandin-resistant isolates, particularly among recurrent infections (3–6). Persistent colonization of *C. auris* on skin and abiotic surfaces has led to multiple outbreaks and poses a risk of invasive disease, including bloodstream infections (7–9). Mortality rates following *C. auris* bloodstream infections have been reported to be 41.7%–47.0% and frequently associated with central venous catheters (7, 10). Given this propensity of central lines as a source for bloodstream infections, recent guidelines have suggested the use of catheter lock solutions as a preventative method in certain high-risk patient populations (11).

Taurolidine, a broad-spectrum antimicrobial with a non-specific mechanism of action exhibiting its effect through interactions with organisms’ cell wall, is utilized in several catheter lock solutions worldwide (12–14). In 2023, a catheter lock solution containing 13,500 mg/L of taurolidine and 1,000 units/mL of heparin (DefenCath) was approved in the USA and represents the first Food and Drug Administration-approved catheter lock solution indicated to reduce the risk of catheter-related bloodstream infections in adult patients with kidney failure receiving chronic hemodialysis through a central line (https://www.fda.gov/drugs/news-events-human-drugs/fda-approves-new-drug-under-special-pathway-patients-receiving-hemodialysis) (12). Taurolidine has been previously tested against *Candida* isolates, including *Candida albicans*, *Candida tropicalis*, *Candida glabrata*, *Candida krusei,* and *C. parapsilosis*, and found to have minimum inhibitory concentration (MIC)_50/90_ values of <250–1,000/250-2,000 mg/L, although no data are available for *C. auris* (15). This study evaluated the *in vitro* activity of taurolidine, the antimicrobial component of the taurolidine/heparin catheter lock solution, against *C. auris*.

A total of 106 clinical isolates (Table S1) were obtained from multiple sources across the USA, Europe, and Africa (16). Sixty four percent of isolates were collected after 2018 and were mostly from bloodstream infections [82% (84 of 106)]. Fungal species identification was confirmed using matrix-assisted laser desorption ionization-time of flight mass spectrometry. Fungal isolates were tested for antimicrobial activity using the broth microdilution methodology per Clinical and Laboratory Standards Institute (CLSI) guidelines, including quality control (QC) strains (17). *C. auris* isolates were stored frozen and subcultured once before *in vitro* testing to minimize passage of clinical isolates. MIC values were read after 24 hours of incubation. Because MIC reading criteria have not been established for taurolidine, all MIC values were read at both 50% and 100% growth inhibition. Caspofungin and fluconazole were read at 50% inhibition, and amphotericin B was read at 100% inhibition. MIC values were validated by concurrently testing CLSI-recommended QC strains, *C. krusei* ATCC 6258 and *C. parapsilosis* ATCC 22019 (18). All MIC values for QC strains were within the acceptable QC ranges as published by CLSI (18). The tentative resistant (*R*) Centers for Disease Control and Prevention (CDC) breakpoints were amphotericin B (*R* ≥ 2 mg/L), caspofungin (*R* ≥ 2 mg/L) and fluconazole (*R* ≥ 32 mg/L) (https://www.cdc.gov/candida-auris/hcp/laboratories/antifungal-susceptibility-testing.html?CDC_AAref_Val=https://www.cdc.gov/fungal/candida-auris/c-auris-antifungal.html).

Taurolidine exhibited antimicrobial activity against all tested *C. auris* isolates with MIC values of ≤1,024 mg/L, whether read at 50% or 100% inhibition. The MIC_50/90_ values were similar when using 50% inhibition (MIC_50/90_, 256/512 mg/L) or 100% reading criterion (MIC_50/90_, 512/512 mg/L; Table 1). Taurolidine activity was similar for all *C. auris* subsets tested regardless of source. All MIC values observed were within one doubling dilution of the modal MIC value 512 mg/L when read at 100% inhibition (Table S1). When applying the tentative CDC MIC breakpoints: 57.5% of *C. auris* isolates (*n* = 61, MIC ≥2 mg/L) were resistant to amphotericin B; 3.7% (*n* = 3, MIC ≥2 mg/L) were resistant to caspofungin; and 86.8% (*n* = 92, MIC ≥32 mg/L) were resistant to fluconazole (Table 1). However, because these isolates were not randomly selected, these resistance rates should not be extrapolated to represent all *C. auris* isolates. Taurolidine activity was unaffected by resistance to amphotericin B, caspofungin, or fluconazole as similar MIC_50/90_ values were observed for all three resistant subsets (Table 2).

**TABLE 1:** Cumulative distributions of MIC values for taurolidine and comparators tested against *Candida auris*

Antimicrobial agent (no. of isolates)	No. and cumulative % of isolates inhibited at MIC (mg/L) of																	
	0.06	0.12	0.25	0.5	1.0	2.0	4.0	8.0	16.0	32.0	64.0	128.0	256.0	512.0	1,024.0	>^*a*^	MIC_50_	MIC_90_
Taurolidine 50% inhibition (106)										0.0 0.0	1.0 0.9	12.0 12.3	58.0 67.0	35.0 100.0			256	512
Taurolidine 100% inhibition (106)												0.0 0.0	14.0 13.2	89.0 97.2	3.0 100.0		512	512
Amphotericin B (106)			0.0 0.0	13.0 12.3	32.0 42.5	59.0^*b*^ 98.1	0.0 98.1									2.0 100.0	2	2
Caspofungin (81)	15.0 18.5	35.0 61.7	20.0 86.4	7.0 95.1	1.0 96.3	0.0 96.3	0.0 96.3	0.0 96.3								3.0 100.0	0.12	0.5
Fluconazole (106)					0.0 0.0	1.0 0.9	9.0 9.4	3.0 12.3	1.0 13.2	4.0 17.0	17.0 33.0					71.0 100.0	>64	>64

^*a*^ Greater than the highest concentration tested.

^*b*^ Shaded values represent resistant isolates according to CDC tentative MIC breakpoints published at https://www.cdc.gov/candida-auris/hcp/laboratories/antifungal-susceptibility-testing.html?CDC_AAref_Val=https://www.cdc.gov/fungal/candida-auris/c-auris-antifungal.html.

**TABLE 2:** Cumulative distributions of MIC values for taurolidine tested against comparator-resistant *Candida auris* subsets

Resistant ^*a*^ subset (no. of isolates)	No. and cumulative % of isolates inhibited at MIC (mg/L) of:								MIC_50_	MIC_90_
Antimicrobial agent	16	32	64	128	256	512	1,024	> ^*b*^		
Amphotericin B-resistant isolates (61)										
Taurolidine 50% inhibition			0.0 0.0	1.0 1.6	37.0 62.3	23.0 100.0			256	512
Taurolidine 100% inhibition				0.0 0.0	1.0 1.6	57.0 95.1	3.0 100.0		512	512
Caspofungin-resistant isolates (3)										
Taurolidine 50% inhibition				0.0 0.0	2.0 66.7	1.0 100.0			256	–
Taurolidine 100% inhibition					0 0.0	3.0 100.0			512	–
Fluconazole-resistant isolates (92)										
Taurolidine 50% inhibition		0.0 0.0	1.0 1.1	11.0 13.0	48.0 65.2	32.0 100.0			256	512
Taurolidine 100% inhibition				0.0 0.0	12.0 13.0	77.0 96.7	3.0 100.0		512	512

^*a*^ CDC tentative MIC breakpoints published at https://www.cdc.gov/candida-auris/hcp/laboratories/antifungal-susceptibility-testing.html?CDC_AAref_Val=https://www.cdc.gov/fungal/candida-auris/c-auris-antifungal.html.

^*b*^ Greater than the highest concentration tested.

The taurolidine MICs reported here are consistent with previous findings for *Candida* spp. and for the mycelial fungal pathogen, *Mucor* spp. (15, 19). When considering the MICs, one would generally relate these values to clinically available breakpoints. However, given taurolidine’s use as a catheter lock solution and not as a systemic therapeutic, breakpoints are not available. Instead, these MICs would most appropriately be evaluated relative to the concentration utilized within the catheter lock solution. As noted above, the combination of taurolidine and heparin approved in the USA contains 13,500 mg/L of taurolidine, and concentrations of 13,500 or 20,000 mg/L are available in catheter lock solutions outside the USA. As such, concentrations within the lumen of a catheter locked with one of the commercially available taurolidine-containing solutions would be ~13- to 20-fold higher than the upper end of the MIC distribution. While the pharmacodynamics of taurolidine have not been described, a single case report suggests the ability of taurolidine, in catheter lock solution, to prevent further bloodstream infections in a 3-year-old child with a history of recurrent *C. glabrata* catheter-related infections (20). As expected, taurolidine was more potent using the 50% inhibition reading criterion as compared with 100%. That said, the MIC distribution remained quite narrow with only a slight shift in distribution toward higher MICs at the higher reading criterion (i.e., MIC_50_ of 256 mg/L vs 512 mg/L) and only three isolates extending the range to 1,024 mg/L. Though the sample size of each group was small, taurolidine activity appeared unaffected by the geographic origin or isolate clade.

The data generated herein support the broad-spectrum activity of taurolidine against *C. auris*, with MIC values well below concentrations utilized in commercially available catheter lock solutions. Given the recent observation of *C. auris* outbreaks and the association with central venous catheters, these data provide the theoretical basis for anticipating prevention of *C. auris* catheter-related bloodstream infection using taurolidine-based catheter lock solutions. Future studies could evaluate these *in vitro* findings in animal and/or clinical studies.

## SUPPLEMENTAL MATERIAL

Table S1 aac.00381-24-s0001.pdf MIC values for strains and isolates tested in this study.

ASM does not own the copyrights to Supplemental Material that may be linked to, or accessed through, an article. The authors have granted ASM a non-exclusive, world-wide license to publish the Supplemental Material files. Please contact the corresponding author directly for reuse.
