# Supplementary material for: In vitro activity of taurolidine against clinical Candida auris isolates: relevance to catheter-related bloodstream infections
Source: Antimicrob Agents Chemother. 2024 Jun 12;68(7):e00381-24. doi: 10.1128/aac.00381-24 (PMC11232392; doi:10.1128/aac.00381-24)
Supplement: Table S1 — MIC values for strains and isolates tested in this study. [file aac.00381-24-s0001.pdf]

Supplemental Table MIC values for strains and isolates tested in this study

Isolate No.	Source	Alias	Study Year	Country	Specimen / Infection type	Clade	Age	Gender	MIC (mg/L) Taurolidine (50% Read)	Taurolidine (100% Read)	Amphotericin B	Caspofungin	Fluconazole
1	SENTRY	--	2013	USA	Blood culture	--	60	F	256	512	2	0.12	>64
2	SENTRY	--	2015	USA	Blood culture	--	61	M	512	512	1	0.5	>64
3	SENTRY	--	2016	USA	Blood culture	--	85	F	512	512	1	0.25	>64
4	SENTRY	--	2016	USA	Blood culture	--	79	M	512	512	1	0.12	>64
5	SENTRY	--	2017	USA	Blood culture	--	69	M	256	512	2	0.12	>64
6	SENTRY	--	2018	USA	Blood culture	--	63	M	512	512	2	0.12	>64
7	SENTRY	--	2018	USA	Blood culture	--	68	M	512	512	2	0.12	>64
8	SENTRY	--	2018	USA	Blood culture	--	77	M	512	512	2	0.12	>64
9	SENTRY	--	2019	USA	Blood culture	--	61	M	512	512	2	0.03	>64
10	SENTRY	--	2019	USA	Blood culture	--	48	F	256	512	2	0.12	>64
11	SENTRY	--	2020	USA	Tissue	--	83	M	256	512	1	0.5	4
12	SENTRY	--	2020	USA	BL/W	--	58	M	256	512	2	0.12	>64
13	SENTRY	--	2020	USA	BL/W	--	82	M	256	512	2	0.12	>64
14	SENTRY	--	2021	USA	Blood culture	--	72	M	256	512	2	0.12	32
15	SENTRY	--	2021	USA	BSI	--	54	F	256	512	2	0.12	>64
16	SENTRY	--	2021	USA	BSI	--	76	M	256	512	2	0.12	>64
17	SENTRY	--	2022	USA	Blood culture	--	61	M	256	512	2	0.12	>64
18	SENTRY	--	2022	USA	W/D/U	--	65	M	128	256	0.5	0.12	>64
19	SENTRY	--	2022	USA	BSI	--	64	F	256	512	2	0.12	64
20	SENTRY	--	2022	USA	W/D/U	--	25	M	128	256	0.5	0.12	>64
21	SENTRY	--	2022	USA	W/D/U	--	56	M	128	256	1	0.12	>64
22	SENTRY	--	2022	USA	W/D/U	--	25	M	128	256	0.5	0.12	>64
23	SENTRY	--	2022	USA	W/D/U	--	37	M	128	256	0.5	0.12	>64
24	SENTRY	--	2022	USA	W/D/U	--	89	F	256	512	2	0.12	>64
25	SENTRY	--	2022	USA	Blood culture	--	59	M	256	512	2	0.12	>64
26	SENTRY	--	2022	USA	--	--	--		128	256	0.5	1	>64
27	SENTRY	--	2009	Germany	Blood culture	--	53	M	256	512	2	0.5	>64
28	SENTRY	--	2020	Greece	Blood culture	--	--	F	512	512	2	0.25	16
29	SENTRY	--	2020	Greece	Blood culture	--	--	F	512	512	1	0.25	64
30	SENTRY	--	2020	Greece	Blood culture	--	--	M	512	512	2	0.25	64
31	SENTRY	--	2020	Greece	Blood culture	--	--	F	512	512	2	0.25	64
32	SENTRY	--	2020	Greece	Blood culture	--	--	M	512	512	2	0.5	64
33	SENTRY	--	2021	Greece	Blood culture	--	66	F	256	512	2	0.25	32
34	SENTRY	--	2021	Greece	Blood culture	--	28	F	256	512	2	0.5	32
35	SENTRY	--	2022	Greece	Blood culture	--	86	F	256	512	2	>8	>64
36	SENTRY	--	2022	Greece	Blood culture	--	63	M	256	512	2	0.5	>64
37	SENTRY	--	2022	Greece	Blood culture	--	60	F	256	512	2	0.25	64
38	SENTRY	--	2022	Greece	Blood culture	--	55	M	256	512	2	0.5	64
39	SENTRY	--	2022	Greece	Blood culture	--	77	M	512	512	2	>8	>64

Isolate No.	Source	Alias	Study Year	Country	Specimen / Infection type	Clade	Age	Gender	MIC (mg/L) Taurolidine (50% Read)	Taurolidine (100% Read)	Amphotericin B	Caspofungin	Fluconazole
40	SENTRY	--	2022	Israel	Blood culture	--	77	F	64	256	1	0.12	>64
41	SENTRY	--	2022	Romania	BSI	--	--	--	256	512	2	0.25	>64
42	SENTRY	--	2022	Romania	BSI	--	--	--	256	512	2	0.12	>64
43	SENTRY	--	2022	Romania	BSI	--	--	--	256	512	2	0.12	>64
44	SENTRY	--	2022	Romania	BSI	--	--	--	256	512	2	0.12	>64
45	SENTRY	--	2022	Romania	BSI	--	--	--	512	512	2	0.25	>64
46	SENTRY	--	2022	Romania	BSI	--	--	--	256	512	2	0.25	>64
47	SENTRY	--	2022	Romania	BSI	--	--	--	256	512	2	0.12	>64
48	SENTRY	--	2022	Romania	BSI	--	--	--	512	512	2	0.25	>64
49	SENTRY	--	2022	Romania	BSI	--	--	--	256	512	2	0.25	>64
50	SENTRY	--	2022	Romania	BSI	--	--	--	256	512	2	0.25	>64
51	SENTRY	--	2022	Turkey	BSI	--	58	M	256	512	2	0.25	64
52	SENTRY	--	2022	Turkey	BSI	--	67	M	512	512	2	0.25	64
53	SENTRY	--	2022	Turkey	Blood culture	--	77	F	256	512	2	0.12	64
54	SENTRY	--	2022	Turkey	Blood culture	--	82	M	512	512	2	0.25	64
55	SENTRY	--	2022	Turkey	Blood culture	--	50	F	256	512	2	0.25	64
56	SENTRY	--	2022	Turkey	Blood culture	--	77	M	256	512	>4	0.12	>64
57	SENTRY	--	2022	Turkey	Blood culture	--	78	F	512	512	2	0.25	64
58	SENTRY	--	2022	Turkey	Blood culture	--	46	M	512	512	2	0.25	64
59	SENTRY	--	2022	Turkey	Blood culture	--	67	M	512	512	>4	0.03	>64
60	SENTRY	--	2014	Colombia	Blood culture	--	--	M	512	512	1	0.25	64
61	SENTRY	--	2018	Panama	Blood culture	--	65	M	256	512	2	0.06	8
62	SENTRY	--	2018	Panama	Blood culture	--	78	M	512	512	2	0.06	4
63	SENTRY	--	2019	Panama	Blood culture	--	38	M	256	512	2	0.12	64
64	SENTRY	--	2019	Panama	Blood culture	--	76	F	256	512	2	0.12	8
65	SENTRY	--	2020	Panama	BSI	--	58	M	256	512	0.5	0.06	4
66	SENTRY	--	2020	Panama	Blood culture	--	63	M	256	512	0.5	0.06	4
67	SENTRY	--	2021	Panama	BSI	--	58	M	256	512	0.5	0.06	>64
68	SENTRY	--	2021	Panama	BSI	--	62	F	512	512	0.5	0.06	>64
69	SENTRY	--	2022	Panama	Ureteral Catheter	--	48	M	256	512	0.5	>8	>64
70	SENTRY	--	2022	Panama	Blood culture	--	43	F	512	512	1	0.06	>64
71	SENTRY	--	2022	Panama	Blood culture	--	64	F	512	512	0.5	0.06	>64
72	SENTRY	--	2022	Panama	BSI	--	69	M	256	512	0.5	0.12	>64
73	CDC AR	AR Bank# 0385	2012	Venezuela	Blood culture	South America	--	--	512	512	1	--	>64
74	CDC AR	AR Bank# 0386	2012	Venezuela	Blood culture	South America	--	--	512	512	1	--	>64
75	CDC AR	AR Bank# 0931	2013	Venezuela	Blood culture	South America	--	--	512	1024	2	--	>64
76	CDC AR	AR Bank# 1104	--	--	--	South America	--	--	256	512	1	0.12	>64
77	CDC AR	AR Bank# 1105	--	--	--	South America	--	--	256	512	1	0.06	4
78	CDC AR	AR Bank# 0381	2009	Japan	Ear	East Asia	--	--	256	512	1	--	2
79	SENTRY	--	2022	Japan	Blood culture	--	66	F	128	256	1	0.06	>64

Isolate No.	Source	Alias	Study Year	Country	Specimen / Infection type	Clade	Age	Gender	MIC (mg/L)				
									Taurolidine (50% Read)	Taurolidine (100% Read)	Amphotericin B	Caspofungin	Fluconazole
80	Westerdijk	CBS 12372	--	South Korea	Blood culture	--	--	--	256	512	1	--	>64
81	Westerdijk	CBS 12373	--	South Korea	Blood culture	--	--	--	256	512	2	--	>64
82	CDC AR	AR Bank# 1099	--	--	--	East Asia	--	--	256	256	1	0.12	4
83	CDC AR	AR Bank# 1100	--	--	--	East Asia	--	--	128	256	1	0.06	4
84	CDC AR	AR Bank# 1101	--	--	--	East Asia	--	--	128	256	1	0.06	64
85	CDC AR	AR Bank# 0389	2018	India	BAL	South Asia	--	--	512	512	2	--	>64
86	CDC AR	AR Bank# 0390	--	India	Wound	South Asia	--	--	512	512	2	--	>64
87	Westerdijk	CBS 12766	--	India	Blood culture	--	--	--	512	1024	2	--	>64
88	Westerdijk	CBS 12768	--	India	Blood culture	--	--	--	512	1024	2	--	>64
89	CDC AR	AR Bank# 0388	2014	Pakistan	Blood culture	South Asia	--	--	512	512	2	--	>64
90	CDC AR	AR Bank# 0382	2019	Pakistan	Burn wound	South Asia	--	--	256	512	1	--	4
91	CDC AR	AR Bank# 0387	2008	Pakistan	Blood culture	South Asia	--	--	256	512	1	--	4
92	CDC AR	AR Bank# 1097	--	--	--	Iran	--	--	512	512	1	0.03	8
93	Kenya	--	2011	Kenya	Blood culture	--	14	M	256	512	2	--	>64
94	Kenya	--	2012	Kenya	Blood culture	--	16	F	256	512	1	--	>64
95	Kenya	--	2012	Kenya	Blood culture	--	75	M	256	512	1	--	>64
96	Kenya	--	2012	Kenya	Blood culture	--	--	F	256	512	1	--	>64
97	Kenya	--	2012	Kenya	Blood culture	--	56	F	256	512	1	--	>64
98	Kenya	--	2012	Kenya	Blood culture	--	88	F	128	512	2	--	>64
99	Kenya	--	2013	Kenya	Blood culture	--	44	M	256	512	1	--	32
100	Kenya	--	2013	Kenya	Blood culture	--	82	M	256	256	2	--	>64
101	Kenya	--	2013	Kenya	Blood culture	--	71	M	256	512	1	--	>64
102	Kenya	--	2013	Kenya	Blood culture	--	63	M	512	512	1	--	>64
103	CDC AR	AR Bank# 0383	2012	South Africa	Blood culture	Africa	--	--	256	512	1	--	>64
104	CDC AR	AR Bank# 0384	2012	South Africa	Blood culture	Africa	--	--	256	512	1	--	>64
105	CDC AR	AR Bank# 1102	--	--	--	Africa	--	--	128	256	1	0.12	>64
106	CDC AR	AR Bank# 1103	--	--	--	Africa	--	--	128	256	0.5	0.12	>64
